# Single Median Raphe Scrotal incision Orchiopexy: A safe & feasible approach for fixation of Palpable Undescended testes

**DOI:** 10.12669/pjms.37.7.4261

**Published:** 2021

**Authors:** Iftikhar A. Jan, Mokhtar Hassan, Ikram Shalaan, Muna Ahmed Alshehhi

**Affiliations:** 1 Iftikhar A. Jan, Department of Pediatric Surgery, Sheikh Shakhbout Medical City, Abu Dhabi, UAE; 2 Mokhtar Hassan, Department of Pediatric Surgery, Sheikh Shakhbout Medical City, Abu Dhabi, UAE; 3 Ikram Shalaan, Department of Pediatric Surgery, Sheikh Shakhbout Medical City, Abu Dhabi, UAE; 4 Muna Alshehhi, Department of Pediatric Surgery, Sheikh Shakhbout Medical City, Abu Dhabi, UAE

**Keywords:** Palpable testis, Orchidopexy, Trans scrotal orchidopexy, Undescended testes

## Abstract

**Background:**

We wish to share our outcome of single median raphe scrotal incision orchiopexy (SMRSO) regarding safety & feasibility of technique by evaluating ease of access, conversion rate, duration of surgery, success rate, complications, and the need for redo-surgery.

**Methods:**

We retrospectively analyzed data of 277 orchiopexies performed in our department on 224 patients operated on between June 2016 to June 2019. SMRSO was considered for palpable testes that can be brought to the upper limit of the scrotum under anesthesia. The conventional inguinoscrotal approach was used for high lying testes. A median raphe incision was made to access & mobilize the testis on either side, ligation of processus vaginalis performed, and the testes secured in the scrotal pouch. The approach was utilized for both unilateral and bilateral orchiopexies. Follow-up done at one week, one month & six months to evaluate the outcome.

**Results:**

A total of 277 orchiopexies were performed in 224 patients. 237 (86%) orchiopexies were via the median raphe scrotal approach in 184 patients. Out of these, 53 cases had bilateral orchiopexies. 30 (11%) performed via a conventional inguinoscrotal approach and 10 (4%) by laparoscopic approach. The mean duration of surgery for SMRSO was 24 minutes for unilateral and 42 minutes for bilateral cases. Immediate postoperative complications included scrotal hematoma in three (1.6%) cases, Hematoma of the abdominal wall in one case, and scrotal edema in 4 (2 %) patients. All complications were treated conservatively & resolved. No wound infection or testicular atrophy was reported. Long-term complications included testicular ascend in three cases [1.6%].

**Conclusion:**

Single-incision Median Raphe Scrotal orchiopexy is an attractive alternative to the standard inguinoscrotal orchiopexy for palpable low lying undescended testes with a better cosmetic outcome.

## INTRODUCTION

Undescended Testis (UDT) is seen in 2-4% of children at birth, decreasing to about 1% in the first year of life.^1^ Ascending testis is also seen in about 0.5 to 2% of children.^1^ The inguinoscrotal approach is a standard procedure for the fixation of palpable undescended testes.^2^ The inguinal approach for UDT treatment was introduced by Schuller M and Bevan AD in the last century.^3^,^4^ The process requires an inguinal incision to mobilize the testicle, ligation of processus vaginalis, and a transverse scrotal incision for testicular fixation. The approach has been used widely due to its satisfactory outcome. For impalpable testes, a laparoscopic approach is preferred with single or two stages orchiopexy.

Bianchi and Squire introduced a single upper scrotal incision orchiopexy & showed the advantage of shorter operative time, ease of dissection, accelerated healing, less pain, and good cosmetic results.^5^ Some practice the approach; however, it did not gain widespread acceptance because it left two scars in bilateral cases and, in some cases, a noticeable scar in the scrotum. The single incision median raphe scrotal orchiopexy (SMRSO) has the advantage of natural skin crease incision. Due to the scrotal mobility, it is possible to access high lying testicles for mobilization & fixation. It also leads to virtually a scar-less surgery.

We wish to share our experience of SMRSO by evaluating ease of access, conversion rate, duration of surgery, success rate, complications, and the need for redo-surgery.

## METHODS

**Fig.1:**
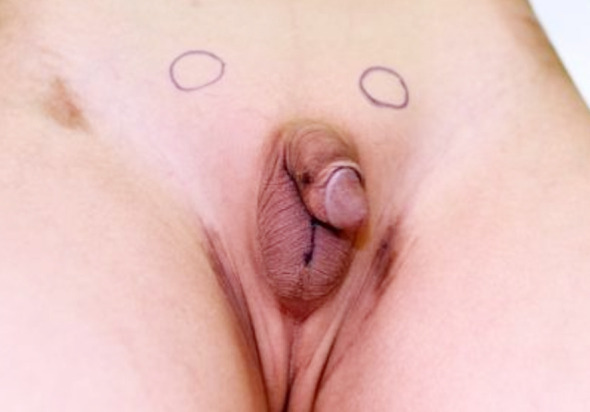
The circles mark the location of both undescended testes.

**Fig.2:**
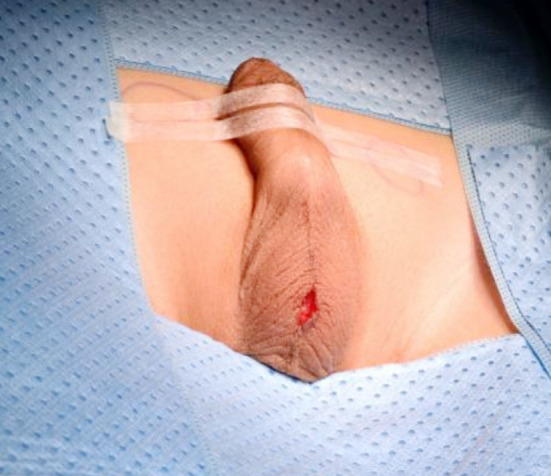
Median Scrotal Incision to access the testes.

**Fig.3:**
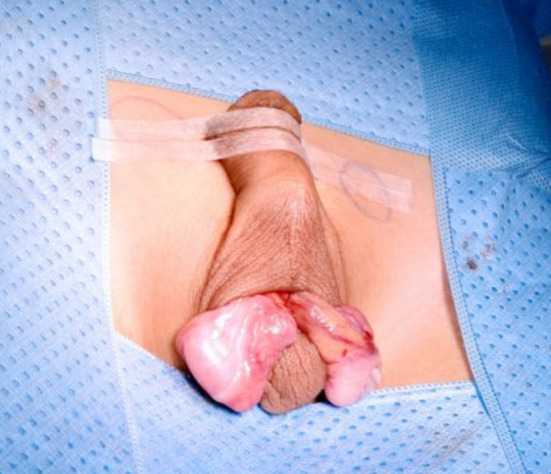
Testes after Mobilization and ligation of Processus vaginalis.

The review was conducted at our department between June 2016 and June 2019 after the ethical committee approval letter no MAFREC-198 dated 09/12/2020. Patients operated for undescended testes (UDT) from six months to 15 years were included in the study. Informed consent was taken from parents for SMRSO with the possibility of conversion to the inguinoscrotal approach. All patients were re-examined after anesthesia induction (Fig.1). The inclusion criteria for SMRSO were a palpable testis that can be pulled down to the upper limit of the scrotum under anesthesia. Testes that could not be brought down were operated by a conventional inguinoscrotal approach with a median raphe scrotal incision. For SMRSO, a median raphe scrotal incision was made, and the affected testis approached through the respective scrotal pouch (Fig.2). Rest of the procedure is similar to the classical orchiopexy except that the whole process is performed through the scrotal approach. It includes the opening of the cremasteric layer, dissection & high ligation of processus vaginalis if present, and two points fixations of testis in the respective hemi-scrotal pouch with absorbable suture (Fig.3). The scrotum pouch & skin was closed with 6/0 absorbable sutures. Caudal block or long-term local anesthesia was used for pain management.

**Fig.4:**
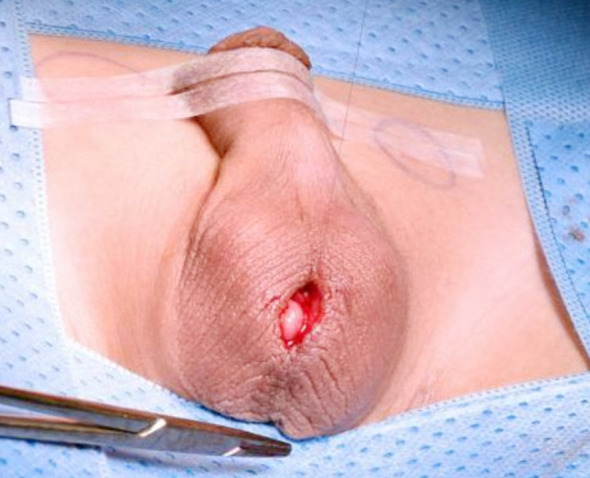
Closure of median Scrotal Incision.

**Fig.5:**
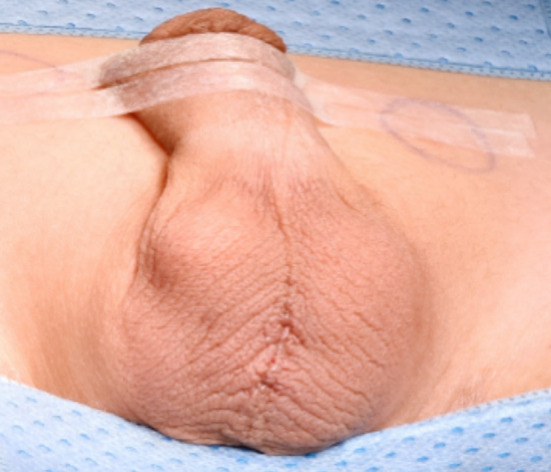
Barely visible surgical wound immediately after skin closure

Testes that remained in the inguinal canal or in the superficial inguinal pouch with difficulty to bring down to the upper scrotum were treated by standard inguinoscrotal approach. Impalpable testes were treated laparoscopically & excluded from the study. In all cases, a median raphe scrotal approach was used for testicular fixation.

## RESULTS

In our series, a total number of 277 orchiopexies were performed in 224 patients. Out of these, 237 (86%) orchiopexies were via the median raphe scrotal approach in 184 patients. Thirty (11%) inguinoscrotal orchiopexies and ten patients (4%) underwent laparoscopic orchiopexy

Among the 184 patients of median raphe scrotal orchiopexy, 68 (36.9%) had left orchiopexy, 63 (34.2%) patients underwent right orchiopexy, and 53 patients (28.8%) underwent bilateral orchiopexy.

A patent processus vaginalis (PPV) was ligated & divided in 70(31 %) patients. Twenty-eight (40%) of the inguinal approach had PPV ligation/ division, and 42(60%) in the scrotal approach group had PPV ligation. In the other patients, the processus vaginalis was not well developed, and an adequate length was achieved for testicular fixation in the scrotum without the need for PPV ligation. In 53 bilateral undescended testes, nine testes were smaller than their counterparts. Eighteen patients had bucket handle epididymis. Three (1.4%) cases required conversion to the inguinoscrotal approach due to difficult mobilization.

In cases treated by an inguinal approach, the testes could be brought comfortably to the scrotum except in two instances where the left testis was fixed to the upper scrotum and later treated with HCG therapy. Operative time ranged between 20 – 49 minutes (mean 24 minutes). The mean operative time in the inguinoscrotal approach was 42 minutes Early Postoperative complications of SMRSO included scrotal hematoma in three cases, Hematoma of the abdominal wall in one case of inguinal scrotal approach & four cases of scrotal edema. All managed conservatively. No wound infection or wound dehiscence reported

Long-term complications included ascending testes in three patients [1.8 %]. Two scrotal approach cases were Ehler Danlos syndrome, and the other was a low-birth-weight ex-preterm baby. The third recurrence was reported with the inguinal approach. No testicular atrophy was reported at follow-up. An excellent scrotal cosmetic result was reported in all patients. High Parent satisfaction of SMRSO was attributed to the pain-free, quick postop recovery & a barely visible scar.

## DISCUSSION

For surgical management, two varieties of UDT are recognized. One palpable undescended testes and other impalpable testes. The conventional surgical approach for palpable undescended testes consists of an inguinal incision, mobilization of the testis, ligation of processus vaginalis, and then fixing the testis through a transverse scrotal incision.^3^ Bianchi & demonstrated the advantages of a single incision scrotal approach.^5^ He used a transverse incision on each hemi-scrotum for scrotal orchiopexy. The transverse scrotal incision has the disadvantage of a visible scar on the scrotum, and two incisions are required for bilateral cases.^5^,^6^ Several surgeons adapted the Bianchi approach and confirmed the safety and efficacy of the scrotal approach with fewer analgesia requirements and early discharge from the Hospital.^7^-^9^ Later, other scrotal techniques were used like the high scrotal approach, low scrotal approach & pre-scrotal approach.^10^,^11^

Median Raphe scrotal orchiopexy is through an existing median raphe scar in the scrotum.^12^ It has several advantages, including easy access, early recovery, minimal postop complications, and virtually scar-less surgery. SMRSO has been used by some authors and reported good results.^12^-^14^ The main concerns with SMRSO are that it may not provide enough access for adequate testes mobilization and may result in a high incidence of recurrence. The other consideration is high ligation of PPV may not be possible and may cause hernia formation. The concerns may look real, but the fact is that the scrotum has very mobile skin. If an appropriate retraction technique is used, it is possible to get high access to the external ring level in children and even up to the internal ring level in infants and babies.^15^,^16^ Bassel YS, et al. showed that PPV ligation may not be required in all cases and does not increase the incidence of a hernia formation after orchiopexy.^8^ Same results were reproduced by Hyuga T et al.^15^ Many surgeons do not routinely perform separation and ligation of PPV and have shown a low rate of inguinal hernia.^8^ We have a similar experience & none of our patients developed an inguinal hernia after scrotal orchiopexy, even though PPV ligation was performed in only 31% of cases. However, we still believe that if a prominent processus vaginalis is present, then the sac shall be dissected and divided after ligation. This will help in reducing the traction by the processus vaginalis and decrease the chances of recurrence.^9^

Poor access was not a concern in our study; only three cases (1.6%) required conversion to an inguinal scrotal approach; thus, it was possible to perform orchiopexy from the scrotal approach in most cases. Studies have shown that 3-5% of patients may require an additional incision from the proper fixation of testis in the scrotum while using a scrotal approach.^16^,^17^ A higher success rate for a pure scrotal approach is possible with appropriate patient selection, and scrotal orchiopexy shall not be attempted with very high lying testes with short pedicles. The best way to avoid failure of SMRSO is an examination under anesthesia, evaluation of the location & mobility of the testis. A scrotal approach may be initiated in doubtful cases & an inguinal incision can be added if required to achieve the desired mobilization and testis fixation without undue traction.

SMRSO has a definite advantage over the inguinoscrotal orchiopexy.^18^ It not only decreases the operative time; it also helps in early recovery, better cosmetic outcome, less risk of wound-related complications, and reduces the cost of surgery. Na SW et al. used a different scrotal approach using a transverse upper scrotal incision for accessing the testis.^19^ Their mean operating time was 18 minutes that is less than our mean operative time (24 minutes). The reason may be that their surgical procedures were done by one senior surgeon, whereas surgeons at various levels of training operated our cases. Mohammad Ramzan M et al. also using a low testicular transverse incision & compared the outcome of the standard versus scrotal orchiopexy approach and found that the mean operating time was 28 minutes with a conversion rate of 7.4%. Our conversion rate was 1.4%, probably due to the selection criteria.^9^

SMRSO, however, is not possible in all cases of undescended testes. Testes lying high in the inguinal canal and with short pedicles may be challenging to mobilize through this approach, and therefore an inguinal incision may be required in these cases. In our department, for all palpable testes below the external ring, we always make a median raphe scrotal incision first. If the testicles cannot be brought down in the scrotum, we add an inguinal incision. Even high lying testicles, especially in the superficial inguinal pouch, often have long pedicle length and can be brought down in the scrotum comfortably through the SMRSO approach. Our low conversion rate of 1.4% supports this technique.

Some authors have used the scrotal approach for recurrent undescended testes & it can be another valuable indication for SMRSO.^20^ We are also using median raphe incision for high testes operated by an inguinal or laparoscopic approach and, in these cases, have the advantage of a barely visible scar. We also use the median raphe incision approach for torsion testes and other testicular conditions and find it very suitable access for most testicular and scrotal conditions.

### Limitations of the study

The end of this approach is impalpable testes and testes lying high in the inguinal canal where an inguinal or laparoscopic approach is required.

## CONCLUSIONS

Median Raphe Scrotal orchiopexy is an attractive alternative to the standard inguinoscrotal orchiopexy. It has a shorter operative time, painless postoperative course, early postoperative recovery, and nearly scarless surgery with an excellent cosmetic outcome. It is a safe and feasible approach for low-lying undescended testes and other conditions requiring testicular access.

### Authors’ Contributions:

**IAJ:** Original concept of procedure. Takes the responsibility and is accountable for all aspects of the work in ensuring that questions related to the accuracy or integrity of any part of the work are appropriately investigated and resolved.

**MH:** Data Collection & manuscript editing.

**IS:** Data Collection.

**MAS:** Contributing surgeon. Manuscript review.
